# Contrasting Trends of Primary Liver Cancer Mortality in Chinese Mongol and Non-Mongol

**DOI:** 10.31557/APJCP.2021.22.9.2757

**Published:** 2021-09

**Authors:** Wen-Qiang He, Xiaoyu Gao, Liqun Gao, Yujia Ma, Dejun Sun, Juan Sun

**Affiliations:** 1 *Inner Mongolia People’s Hospital, Hohhot, Inner Mongolia Autonomous Region, China. *; 2 *University of New South Wales, Sydney, Australia. *

**Keywords:** liver cancer mortality, Mongol, HBV infection, PAF

## Abstract

**Background:**

This study aims to investigate the temporal trend as well as the burden of primary liver cancer among Mongol and non-Mongol in China.

**Materials and Methods:**

The registered data from up to 20 monitoring points in the periods of 2008 to 2015 in Inner Mongolia were used to calculate and model the trend of liver cancer among Mongol and non-Mongol using log-linear regression. Logistic regression was used to characterise the risk of liver cancer by using hospitalization records from 2008 to 2017.

**Results:**

Over the study period, significant reduction of liver cancer mortality was found among non-Mongol population (4.8/100,000 from 23.7/100,000 to 18.9/100,000, p=0.04), while the increase of liver cancer mortality was observed among the Mongolian population (8.4/100,000 from 10.7/100,000 to 19.1/100,000, p=0.02), particularly the Mongol from East (25.5/100,000 from 11.2/100,000 to 36.7/100,000, p=0.005). Comparing to the non-Mongol patients with primary liver cancer, the Mongolian patients were more likely to be from East Inner Mongolia (aOR=3.65, 95% CI:2.75-4.87) and those residing in urban area (aOR=2.11, 95%CI: 1.55-2.91). In 2015, a total of 3056 primary liver cancer deaths could be converted if the four known risk factors (HBV, Hepatitis C Virus, alcohol consumption and smoking) could be prevented. HBV remained to be the leading risk factor of liver cancer (PAF=56%, contributing to 2616 deaths) with the highest among the Mongol from East (PAF=65.1%, contributing to 763 deaths).

**Conclusion:**

The continuing increase of primary liver cancer among Mongol suggested further interventions were needed to combat its burden.

## Introduction

Liver cancer is a common malignant neoplasm with 953,000 incident cases and claiming death toll of 819,000 globally in 2017 (Fitzmaurice et al., 2019). China has accounted for more than half of newly diagnosed liver cancer cases (570,000) in 2017 and it is the third leading cause of cancer deaths with a mortality rate of 26.26 per 100,000 (male: 37.55 and females: 14.45 per 100,000) (Chen et al., 2016), while Mongolia has the highest annual mortality rate of liver cancer globally at 75.4 per 100,000 in 2018 (Bray et al., 2018). The well-known risk factors of liver cancer include infection with hepatitis B virus (HBV), hepatitis C virus (HCV), alcoholic cirrhosis and smoking (Chen and Zhang, 2011). Because of the universal HBV vaccination, chemoprevention in selected population, and early detection in at-risk population (Chen and Zhang, 2011), the reduction of liver cancer incidence and mortality rate was observed in some countries worldwide (Li and He, 2021), including some areas of China in recent years but not Mongolia (Bai et al., 2018; Sun et al., 2018). 

In China, mortality of liver cancer and its temporal trends varied significantly by areas and economic development (Chen et al., 2016; Yang et al., 2017). For instance, one study reported that the liver cancer mortality declined significantly for urban men and urban women while it remained stable for rural men and rural women (Sun et al., 2018). Along with the disparity caused by socioeconomic status, ethnicity was also considered to be an important factor of liver cancer burden (Pham et al., 2018). For instance, the Southeast Asians (Vietnamese, Cambodians, and Laotians) had overall liver cancer incidence rates eight to nine times than non-Hispanic whites and more than twice that of other ethnic Asians in California (Pham et al., 2018). Till now, no study has investigated the burden of liver cancer by ethnicity in China, typically the Mongol.

The Inner Mongolia Autonomous Region is located in northern China and Han and Mongol constitute approximately 96% of the population in 2018 including 4.2 million Mongol accounting for 72% (4.2/5.8 million) of Mongolian population in China, which is the largest Mongolian population in the world (bigger than that in the Republic of Mongolia). Therefore, the study of Mongol in Inner Mongolia could largely represent this population in China. Inner Mongolia is geographically divided into Midwest and East regions due to its long span from west to east (Xin et al., 2014). In 2018, 76% of the Mongol resided in the East of Inner Mongolia (2011). Despite Inner Mongolia is one of the most economic developed provinces in China with annual GDP per capita often ranked 5th in the nation, the two regions (Midwest and East) differ by the economic development. Primary industry is the major economic contributor in the Midwest driven by its rich natural resources (Statistics, 2018). As such, people from Midwest have better access to education and health care system. In contrast, agricultural still plays the main role in the East (Statistics, 2018), where Mongol lead a nomadic existence for millennia with forestry and hunting playing an important part. Therefore, people from East still live relatively poorer with limited public health services.

Although liver cancer ranked 2^nd^ of the most common cancer in Inner Mongolia (Ying et al., 2014), no study has estimated the burden of liver cancer among Mongol and its temporal trend. By using data from consistent Death Registry System (DRS) and hospitalization records, this study aims to understand the burden of primary liver cancer in Mongol and its temporal trend. 

## Materials and Methods

*Study population and definitions*

The mortality data used in this study was collected by Centre for Disease Control and Prevention of Inner Mongolia in real time through an internet-based reporting system, Death Registration System (DRS) since 2008 (Wang et al., 2008). Information on individual deaths in all population catchment areas has been reported using DRS. In this system, information on each death is systematically validated by local – including county, prefecture and provincial level Centers for Disease Control and Prevention, which also checks the completeness, coding and internal logic of the items reported on death certificates. Although previous studies of mortality data have shown under-reporting of deaths, especially among children with the completeness of death registration at 40%, the completeness of death in the population aged 60 years and over was about 80% in 2010 (Guo et al., 2015). According to a recent study, the overall death registration completeness for the death registry in China is 74.2%, while the completeness in Inner Mongolia is slightly lower at 66.0% in 2018 (Zeng et al., 2020). Given the average age at death due to liver cancer at about 60 and all the death registration data from this study have been used by the national study, we assumed at least 80% of completeness was reached for primary liver cancer death in this study. 

The DRS uses a multistage cluster probability sampling strategy based on region distribution (Midwest and East) of Inner Mongolia, the total population of local areas, proportion of rural dwellers and the local domestic product (Xin et al., 2014). A total number of up to 20 monitoring points (County-level divisions) were included from 1 January 2008 to 31 December 2015. From 2008 to 2012, eight monitoring points were included with about 2.4 million population accounting about 10% (2.4 million/24 million) of Inner Mongolian population. They were Linhe, Ejin Horo, Tumd Right, Muslims, Sonid Right in the Midwest and Bairin Right, Kailu, Yakeshi in the East. From 2013 onwards, the monitoring points expended to 20 with about 5.8 million population accounting for 24% (5.8 million/24 million) of Inner Mongolian. The additional 12 counties included 6 in the Midwest (Alxa Left, Haibowan, Dongsheng, Qingshan, Wuchuan, Qahar Right Front) and 6 in the East (Keshiketeng, West Ujimqin, Naiman, Horqin Right Middle, Manzhouli, Arun) (Liu et al., 2016). 

The mortality data collection contains the cause of death according to International Classification of Disease 10th (ICD-10) and demographic information including age, sex, ethnicity, residence, education, etc. The death of liver cancer was found by using ICD-10 code C22 with subcategories from C22.0 to C22.9. ICD-10 code has been implemented in China since 2002 and the accuracy of ICD-10 to detect primary liver cancer has been previously validated with high specificity and acceptable sensitivity (Lapointe-Shaw et al., 2018). Together with mortality data, the CDC of Inner Mongolia collected the number of people residing in the monitoring points annually. The total number of people by region (Midwest and East regions) was estimated by summing the number of people according to their residence. In addition, the total number of people by ethnicity was obtained from Inner Mongolia Census Data (2011). 

Hospitalization records of any patients diagnosed with primary liver cancer were also included in this study from 10 major tertiary hospital in the periods of 2008 to 2017. This included six hospitals in Midwest and four hospitals in the East of Inner Mongolia servicing about 20% of hospitalized patients of the whole region. If the same patient had multiple hospital record of liver cancer either from a single hospital or different hospitals, only the first record was included for this analysis. The medical records included the patient demographic information, medical history, and diagnosis, etc. For this study, the following variables were included in the analysis: age (<=55 years, >55 years), sex (men, women), region (Midwest, East), remoteness of area of residence (rural, urban), occupation (no, yes), medicare (no, yes), year of hospitalization (<=2012, >2012), history of smoking (no, yes) alcohol consumption (no, yes), hepatitis (no, HBV, HCV, HBV/HCV).

*Analysis*

In this study, mortality rate (per 100,000) was calculated by dividing the number of cases over the population overall, as well as by ethnicity and region. Five-year age group (0-85 years with every 5 years as a group) was used for age-specific mortality rate. The 95% confidence interval for mortality rate was calculated by using the normal approximation. The temporal trends in liver cancer mortality by the year of death were then modelled with log-linear regression or linear regression. For the calculation of change over years, we used the first fitted value minus the last fitted value.

To estimate the Population Attributable Fraction (*PAF*), the hospitalization data was used to obtain the prevalence of liver cancer patients (*p*_c_) due to smoking, alcohol drinking, HBV, and HCV infections. Estimates of *PAF* were calculated based on pc and the relative risk (*RR*) of risk factors using the following formula:

$\mathrm{PAF}=p_{c}\frac{\left(\mathrm{RR}-1\right)}{\mathrm{RR}}$

The following estimates of *RR* were used according to a previous study in China (Fan et al., 2013): 18.1 for HBV, 13.1 for HCV, 1.87 for alcohol consumption, 1.36 for smoking. 

To estimate the joined *PAF*, we assumed independence of risk factors including exposure to HBV, HCV, alcohol consumption and smoking. A combined PAF for all risk factors was calculated according to the following formula (Ezzati et al., 2003):

$P\mathrm{AF}=1-\prod_{r=1}^{R}\left(1-{\mathrm{PAF}}_{r}\right)$

where *r *is each individual risk factor, and *R* is the total number of risk factors for liver cancer. The total death attributable to each risk factor as well as the combined factors were calculated by multiplying each *PAF* with the corresponding population in Inner Mongolia.

All analyses were done with *R* version 3.4.2 (Team, 2016). All statistical tests were two sided and a P <0.05 was considered statistically significant. 

## Results

**Figure 1:**
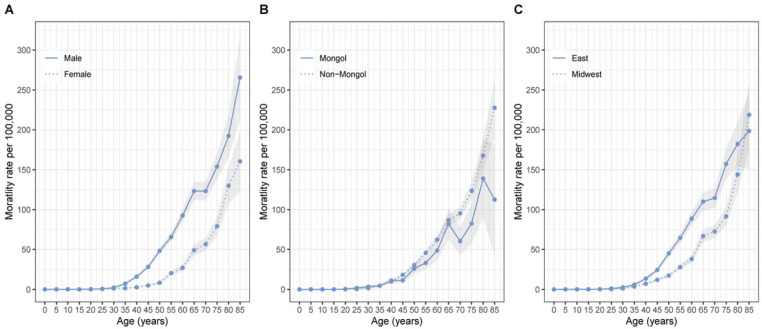
Age-specific Liver Cancer Mortality Rate in Inner Mongolia from 2008 to 2015 by Gender (A), Region of Residence (B) and Ethnicity (C). Connected line represents the age-specific mortality rate and the shaded region represents 95% confidence interval of the mortality rate

Between 1 January 2008 and 31 December 2015, there were 5,428 cases died from liver cancer for people residing in the 20 monitoring points of Inner Mongolia. The study population was characterized by ethnicities including Mongol and non-Mongol. Although Mongol accounted for 9.1% (492/5428) of the primary liver cancer mortality cases, they were more likely to die at younger age than non-Mongol (60.5 vs 62.2, p=0.008). Over the study period, the liver cancer mortality rate was about 18.3 per 100,000 person-years (95% confidence interval [CI], 17.9-18.8). The liver cancer mortality rate increased with age overall and by gender, by ethnicity and by region of residence (Figure 1). Overall, higher mortality rate was found among the male, non-Mongol and those from East Inner Mongolia than the female, Mongol and those from Midwest.

**Figure 2:**
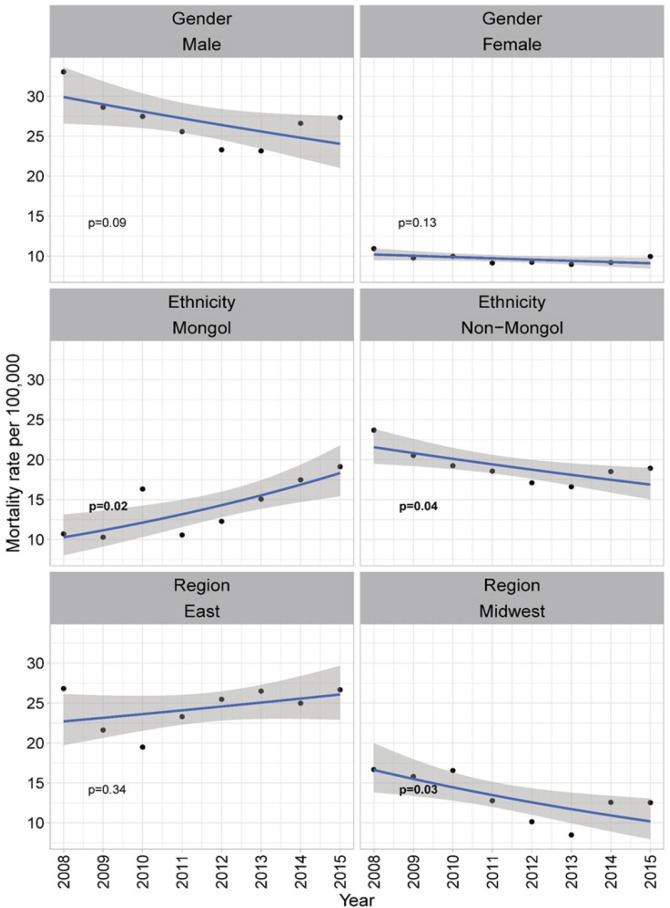
Temporal Trends of Liver Cancer Mortality by Gender, Region of Residence, and Ethnicity. P-value smaller than 0.05 is of significance and in bold

Figure 2 shows the temporal trend of liver cancer mortality by year (Figure 2). Overall, only slight reduction of liver cancer mortality was found from year 2008 to year 2015 (3.3/100,000 from 22.2/100,000 to 18.9/100,000) but without statistical significance (p=0.11). As compared to the reduction among the female (1.0/100,000 from 10.9/100,000 to 9.9/100,000), higher reduction of the liver cancer mortality rate was found among the male (7.6/100,000 from 33.1/100,000 to 25.5/ 100,000, p=0.09). Significant reduction over the study period was found among non-Mongol population (4.8/100,000 from 23.7/100,000 to 18.9/100,000, p=0.04) or those residing in the Midwest (4.2/100,000 from 16.7/100,000 to 12.5/100,000, p=0.03). However, the increase of liver cancer mortality was observed among the Mongolian population (8.4/100,000 from 10.7/100,000 to 19.1/100,000, p=0.02). 

**Figure 3:**
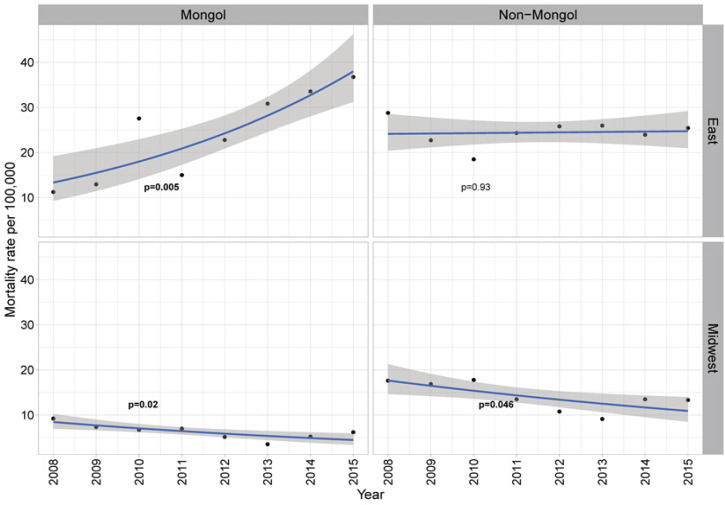
Liver Cancer Mortality Rate in Inner Mongolian Population by Ethnicity and Region of Residence. P smaller than 0.05 is of significance and in bold

Figure 3 shows the temporal trend of mortality by ethnicity and region (Figure 3). From 2008 to 2015, the Mongol from East Inner Mongolia had significant increase of liver cancer mortality (25.5/100,000 from 11.2/100,000 to 36.7/100,000, p=0.005), while mild reduction was observed among Mongol from Midwest (3.0/100,000 from 9.2/100,000 to 6.2/100,000, p=0.02). In contrast, although no significant change was found among non-Mongol in the East (4.3/100,000 from 17.6/100,000 to 13.3/100,000, p=0.046), slight reduction was also observed among those non-Mongol in the Midwest (4.3/100,000 from 17.6/100,000 to 13.3/100,000, p=0.046). 

From 2008 to 2017, a total of 4580 hospitalization cases diagnosed with liver cancer were found from the 10 tertiary hospitals with the Mongol accounting for 6.9% (Table 1). Overall, the Mongolian population with liver cancer were more likely to be from East Inner Mongolia than from Midwest (10.5% vs 3.5%, aOR=3.65, 95% CI:2.75-4.87), those residing in urban area than those in rural area (9.2% vs 3.7%, aOR=2.11, 95%CI: 1.55-2.91), those with occupation than without (7.5% vs 4.2%, aOR=1.55, 95%CI: 1.07-2.30). However, the Mongolian population were less likely to have medicare (11.5% vs 5.4%, aOR=0.41, 95%CI: 0.31-0.54). Although the Mongol with liver cancer also have higher proportion of smoking, drinking alcohol, hepatitis B virus (HBV) infection, no statistical significance was found in this study (smoking: 4.7% vs 8.0%, aOR=1.49, 95% CI: 0.86-2.58; alcohol consumption: 4.7% vs 8.2%, aOR=1.41, 95% CI: 0.81-2.45; HBV: 6.3% vs 7.6%, aOR=0.89, 95% CI: 0.66-1.23). 

Table 2 shows the PAF of liver cancer attributable to HBV, HCV, smoking, and alcohol drinking as well as the total number of deaths could be reduced if the risk factor could be eliminated in 2015. About 65.5% of liver cancer death were attributed to the four risk factors combined and a total of 3056 death cases were caused by them. However, the combined PAF for the Mongol from East was as high as 75.9% (contributing to 889 deaths). Of the four risk factors, HBV remained to be the leading risk factor of liver cancer (PAF=56%) with the highest among the Mongol from East (PAF=65.1%). Compared to the non-Mongol, the Mongol had higher PAF due to smoking and alcohol consumption. 

**Table 1:** Multivariate Analysis of Liver Cancer Hospitalization by Ethnicity in Inner Mongolia, 2008-2015

	Mongol/All (%)	aOR (95% CI)	P value
Overall	315/4580 (6.9)		
Age			
<=55	116/1537 (7.5)	Ref	
55+	199/3040 (6.5)	0.87 (0.67, 1.14)	0.31
Sex			
Men	232/3534 (6.6)	Ref	
Women	83/1036 (8)	1.37 (1, 1.85)	0.05
Region			
Midwest	86/2481 (3.5)	Ref	
East	204/1941 (10.5)	3.65 (2.75, 4.87)	<0.001
Address of residence			
Rural	58/1582 (3.7)	Ref	
Urban	224/2428 (9.2)	2.11 (1.55, 2.91)	<0.001
Unknown	33/570 (5.8)		
Occupation			
No	38/909 (4.2)	Ref	
Yes	270/3594 (7.5)	1.56 (1.08, 2.3)	0.02
Unknown	7/77 (9.1)		
Medicare			
No	140/1221 (11.5)	Ref	
Yes	154/2855 (5.4)	0.41 (0.31, 0.54)	<0.001
Unknown	21/504 (4.2)		
Year of hospitalization			
<=2012	108/1342 (8)	Ref	
2012+	206/3237 (6.4)	0.84 (0.64, 1.12)	0.23
Smoking			
No	116/2462 (4.7)	Ref	
Yes	68/855 (8)	1.49 (0.86, 2.58)	0.15
Unknown	131/1263 (10.4)		
Alcohol consumption			
No	120/2561 (4.7)	Ref	
Yes	67/816 (8.2)	1.41 (0.81, 2.45)	0.22
Unknown	128/1203 (10.6)		
Hepatitis			
No	81/1278 (6.3)	Ref	
HBV	158/2070 (7.6)	0.89 (0.66, 1.23)	0.48
HCV	11/163 (6.7)	0.45 (0.19, 0.93)	0.04
HBV/HCV	3/29 (10.3)	1.49 (0.34, 4.58)	0.54
Unknown	62/1040 (6)		

HBV, hepatitis B virus; HCV, hepatitis C virus.

**Table 2:** Number of Primary Liver Cancer Deaths in 2015 and Fraction Attributable to HBV, HCV, Alcohol Consumption and Smoking Overall and by Ethnicity

	HBV		HCV		Alcohol		Smoking		Combined	
	PAF (%)	Deaths	PAF (%)	Deaths	PAF (%)	Deaths	PAF (%)	Deaths	PAF (%)	Deaths
Mongol										
East	65.1	763	5.4	64	18.4	216	10.6	124	75.9	889
Midwest	52.6	33	1.5	1	17.7	11	10.9	7	65.8	41
Non-Mongol										
East	59.6	1553	9	233	12.5	326	7.1	185	70.1	1826
Midwest	52.6	718	1.5	20	10.2	139	6.5	88	60.8	829
Overall	56	2616	5	233	11.3	526	6.8	319	65.5	3056

PAF, population attributable fraction; HBV, hepatitis B virus; HCV, hepatitis C virus.

## Discussion

In this study, we found the average mortality rate of liver cancer in Inner Mongolia was about 18.3 per 100,000 person-years from 2008 to 2015. Although some reduction of primary liver cancer was found among non-Mongol, a significant increase of liver cancer mortality (5.2 per 100,000) was found among the Mongolian population, particularly the Mongolian population in the East with an increase of 16.5 per 100,000. About 76% of primary liver cancer deaths among them were attributable to the four combined risk factors (primarily HBV contributing to 65%), equally, about 900 deaths could be converted if all these risk factors were eliminated. Therefore, strategies should be taken to address the increasing burden of liver cancer mortality among the Mongol in the East. 

Our study had found opposite trends of liver cancer mortality by ethnicity with significant increase among the Mongol and some reduction among non-Mongol. This is in line with other reports with varied trends of liver cancer mortality rate in China. Although the national mortality of liver cancer decreased slightly from 22 population-based Chinese cancer registries from 2000 to 2011 (Chen et al., 2016), significant decline in liver cancer mortality was only observed among urban male and female but the liver cancer mortality remained stable for rural male and female from 1991 to 2014 (Sun et al., 2018). The disparity of liver cancer mortality trends between urban and rural residents can be attributed to the unequal medical levels and resources (Sun et al., 2018). This could also be attributed to disparities in HBV vaccination coverage in China Centers for Disease Control and Prevention (CDC), 2007, as HBV vaccine was not included in the national vaccination program until 2003 and children from provinces with higher income had substantially higher coverage than those from provinces with lower income Centers for Disease Control and Prevention (CDC), 2007. 

To our knowledge, no study has reported the burden of primary liver cancer among Mongol in China. Primarily, the increasing mortality rate among them were driven by those Mongol residing in the Eastern Inner Mongolia as slight reduction was found among Mongol from Midwest. Our estimates indicated that the liver cancer mortality rate among Mongol from East was higher than the national level in 2015 (36 per 100,000 vs 26 per 100,000), while the mortality rate from the Midwest (5 per100,000) was much lower than the national level (Chen and Zhang, 2011). This large variation could be associated with difference of the socioeconomic development between these two regions in Inner Mongolia, as the Midwest of Inner Mongolia is considered more developed than the East of Inner Mongolia, which could provide better health care system, medication, and treatment (Statistics, 2018). This is supported by previous studies with higher mortality rate of both pancreatic cancer and intracerebral hemorrhage from East Inner Mongolia than that from Midwest (Xiaoyan Zhang, 2017; Gao et al., 2019). Apart from the difference of socioeconomic development, the ethnicity could also contribute to the difference. As reported in previous study that Southeast Asians had eight to nine times of liver cancer incidence rates than that among non-Hispanic whites and more than twice that of other ethnic Asians in California (Pham et al., 2018).

The continuing high burden of liver cancer among Mongol in the East might also contribute to the exposure to the well-known risk factors, including infection with HBV, HCV, alcohol consumption, dietary aflatoxins, and tobacco smoking (Chen et al., 2010; Khazaei et al., 2018). In this study, the Mongol in the East had the highest attributable fraction of HBV (65%) than other groups, which was similar to the PAF reported previously from previous national study at 64% (Fan et al., 2013). However, the PAF from the previous study was based on the national prevalence of HBV from a cross-sessional sero-epidemiologic study in 1992 and the number of liver cancer deaths in 2005 (Fan et al., 2013). Our study utilized the hospitalization record to obtain the proportion of cases exposed would thus provide more accurate and up to date estimate of PAF. In addition to the HBV infection, this study also found higher PAF among Mongol due to smoking and alcohol drinking. This is in line with their food consumption as Mongol tend to drink more strong wine, consume fewer fresh vegetables (Fu et al., 2000; Zhai et al., 2007). Based on our calculation, we propose about 900 liver cancer deaths among Mongol in 2015 could be prevented if all these risk factors could be modified. Therefore, further intervention study is needed to validate the effect of these strategies to reduce the burden of liver cancer.

The strength of this study is using consistent administrative data to calculate the temporal trends of primary liver cancer among Mongol and non-Mongol. Furthermore, the comprehensive hospitalization data allows us to characterize the population with primary liver cancer, which also provides us the chance to estimate PAF due to HBV, HCV, smoking, and alcohol. The main limitation of this study is that we could not estimate the incidence of liver cancer due to the lack of incidence data. However, based on the mortality and hospitalization data, we propose the incidence of liver cancer in Inner Mongolia is lower than the national level and the incidence among the Mongolian population will continue to increase. The other limitation is the completeness and validity of data used in this study. As for death registration data, the completeness was about 80% among people aged 60 years or above in 2010 and it has improved over the study period in China (Guo et al., 2015; Zeng et al., 2020). Therefore, the yearly liver cancer mortality might be underestimated in this study. However, the trend over the study period would not be affected as it was generally consistent with national report from China (Chen et al., 2016; Yang et al., 2017). As for the hospital data, the incomplete hospitalization information was due to the electronic medical records only started from 2011 and the implementation varied by hospitals. However, we assure our estimates were accurate as the findings were consistent with the mortality data. In addition, the hospitalization data could also not be linked to the death records. As such the survival of liver cancer was unable to calculate and whether the trend of survival changed over time remained unknown. Further study should try to link the hospitalization data to the mortality data. Therefore, we will be able to estimate the reduction of liver cancer mortality in the non-Mongol is due to the increased survival rate or decreased incidence. Lastly, according to previous study in China, aflatoxin exposure contributed to about 25% of primary liver cancer (Liu et al., 2012). The lack of aflatoxin exposure in this study resulted in our highest combined PAF at about 75%, which was about 10% lower than the previous study (Fan et al., 2013). 

In conclusion, this study showed that the increasing mortality of primary liver cancer among Mongol, particularly those residing in the East Inner Mongolia, while reduction was found in the more developed Midwest of Inner Mongolia. We estimated that about 900 Mongolian deaths could be reduced if the known risk factors were able to be prevented. Our results highlighted interventions are needed to prevent the continuing increasing burden of primary liver cancer among Mongol.

## Author Contribution Statement

Conceptualization: WH, DS, JS; Data acquisition: DS, JS; Formal analysis: WH; Writing-original draft: WH; Reviewing: XG, LG, YM, DS, JS. 
